# *PiiL*: visualization of DNA methylation and gene expression data in gene pathways

**DOI:** 10.1186/s12864-017-3950-9

**Published:** 2017-08-02

**Authors:** Behrooz Torabi Moghadam, Neda Zamani, Jan Komorowski, Manfred Grabherr

**Affiliations:** 1 Department of Cell and Molecular Biology, Computational and Systems Biology, Uppsala University, Uppsala, Sweden; 2 Department of Medical Biochemistry and Microbiology/BILS, Genomics, Uppsala University, Uppsala, Sweden; 3 Department of Plant Physiology, Umeå University, Umeå, Sweden; 4 Institute of Computer Science, Polish Academy of Sciences, 01248 Warsaw, Poland

**Keywords:** DNA methylation, Gene expression, Visualization, KEGG pathways

## Abstract

**Background:**

DNA methylation is a major mechanism involved in the epigenetic state of a cell. It has been observed that the methylation status of certain CpG sites close to or within a gene can directly affect its expression, either by silencing or, in some cases, up-regulating transcription. However, a vertebrate genome contains millions of CpG sites, all of which are potential targets for methylation, and the specific effects of most sites have not been characterized to date. To study the complex interplay between methylation status, cellular programs, and the resulting phenotypes, we present *PiiL*, an interactive gene expression pathway browser, facilitating analyses through an integrated view of methylation and expression on multiple levels.

**Results:**

*PiiL* allows for specific hypothesis testing by quickly assessing pathways or gene networks, where the data is projected onto pathways that can be downloaded directly from the online KEGG database. *PiiL* provides a comprehensive set of analysis features that allow for quick and specific pattern searches. Individual CpG sites and their impact on host gene expression, as well as the impact on other genes present in the regulatory network, can be examined. To exemplify the power of this approach, we analyzed two types of brain tumors, Glioblastoma multiform and lower grade gliomas.

**Conclusion:**

At a glance, we could confirm earlier findings that the predominant methylation and expression patterns separate perfectly by mutations in the *IDH* genes, rather than by histology. We could also infer the IDH mutation status for samples for which the genotype was not known. By applying different filtering methods, we show that a subset of CpG sites exhibits consistent methylation patterns, and that the status of sites affect the expression of key regulator genes, as well as other genes located downstream in the same pathways.

*PiiL* is implemented in Java with focus on a user-friendly graphical interface. The source code is available under the GPL license from https://github.com/behroozt/PiiL.git.

**Electronic supplementary material:**

The online version of this article (doi:10.1186/s12864-017-3950-9) contains supplementary material, which is available to authorized users.

## Background

DNA methylation (DNAm) is a key element of the transcriptional regulation machinery. By adding a methyl group to CpG sites in the promoter of a gene, DNAm provides a means to temporarily or permanently silence transcription [1], which in turn can alter the state or phenotype of the cell. DNAm of sites outside promoters can also take effect, where for example methylation in the gene body might elongate transcription, and methylation of intergenic regions can help maintain chromosomal stability at repetitive elements [2]. Change in DNAm has been observed to occur with age in the human brain [3, 4], as well as in various developmental stages [5]. It is also a hallmark of a number of diseases [6, 7], including cancer [8, 9]. A prominent example is the methylation of the promoter of the tumor suppressor protein *TP53* [10–12], which occurs in about 51% of ovarian cancers [13]. Since *TP53* is a master regulator of cell fate, including apoptosis, disabling its expression has a direct impact on the function of downstream expression pathways.

Different cancers or cancer subtypes, however, might deploy different strategies to alter expression patterns to increase their viability, which might be visible in the methylation landscape. In gliomas, for instance, it has been reported that mutations in the IDH (isocitrate dehydrogenase genes 1 and 2, collectively referred to as IDH) genes result in the hyper-methylation of a number of sites [14].

However, with a few exceptions, the exact relation between DNA methylation and the expression of its host gene remains elusive and is still poorly understood. One confounding factor is the many-to-one relationship between CpG sites and genes or transcripts. A global association of lower expression with increased promoter methylation, and increased expression with methylation of sites in the gene body has been observed [2, 15–17]. By contrast, an accurate means to predict the effect of methylating or de-methylating any given site, or clusters thereof, is still lacking. In addition, altering the expression of certain genes might not be relevant for disease progression but rather becomes a side effect, whereas changes in key regulators of networks might result in large-scale effects. Characterizing the methylation patterns that differ between tumor types allows for a more accurate diagnosis and can thus inform the choice of treatment. Moreover, examining the effect on the regulatory machinery in a pathway or gene expression network level might give insight into how the disease develops, progresses, and spreads [18].

Here, we present *PiiL* (*P*athway *i*nteractive v*i*sualization too*l*), an integrated DNAm and expression pathway browser, which is designed to explore and understand the effect of DNAm operating on individual CpG sites on overall expression patterns and transcriptional networks. *PiiL* implements a multi-level paradigm, which allows examining global changes in expression, comparisons between multiple sample grouping, play-back of time series, as well as analyzing and selecting different subsets of CpG sites to observe their effect. Moreover, *PiiL* accepts pre-computed sub-sets that were generated offline by other methods, for example the *bumphunter* function in Minfi [19], Monte Carlo Feature Selection (MCFS) [20], or unsupervised methods, such as Saguaro [21]. *PiiL* accesses pathways or gene networks online from the KEGG databases [22, 23], and allows for visualizing pathways from different organisms with up-to-date KEGG pathways.

In keeping a sharp focus on methylation, expression, and ease-of-use, *PiiL* builds upon the user experience with other, typically more general visualization tools. For example, Cytoscape [24] is a widely-used, open source platform for producing, editing, and analyzing generic biological networks. The networks are dynamic and can be queried and integrated with expression, protein-protein interactions data, and other molecular states and phenotypes, or be linked to functional annotation databases. Due to the extensibility of the core, there are multiple plugins available, some specifically for handling KEGG databases, such as KEGGscape [25] and CyKEGGParser [26], features that are natively built into *PiiL*. Pathview [27], an R/Bioconductor package, also visualizes KEGG pathways with a wide range of data integration, such as gene expression, protein expression, and metabolite level on a selected pathway, but, unlike *PiiL*, lacks the ability to examine methylation at the resolution of individual sites. Pathvisio [28], another tool implemented in Java, provides features for drawing, editing, and analyzing biological pathways, and mapping gene expression data onto the targeted pathway. Extended functionality is added via different available plugins, but similar to Pathview, it does not provide functionality specific to analyze the effects of DNAm based on individual sites. KEGGanim [29] is a web-based tool that can visualize data over a pathway and produce animations using high-throughput data. KEGGanim thus highlights the genes that have a dynamic change over conditions and influence the pathway, a feature that is also available in *PiiL*.

In the following, we will first describe the method, and then exemplify how *PiiL* benefits the analysis of large and complex data sets without requiring the user to be an informatics expert.

## Implementation

*PiiL* is platform independent, implemented in Java with an emphasis on user-friendliness for biologists. It first reads KGML format pathway files, either from a storage media, or from the online KEGG database (using REST-style KEGG API), where in case of the latter, a complete list of available organisms and available pathways for the selected organism is loaded and locally cached for the current session. Multiple pathways can be viewed in different tabs, with each tab handling either DNAm or gene expression data, referred to as metadata in this article.

According to the metadata, genes are color-coded based on individual samples, or a user-defined grouping. The user can also load a list of genes with no metadata, and find overlapping genes highlighted in the pathway of interest.

### Obtaining information about the pathway elements

Gene interactions (activation, repression, inhibition, expression, methylation, or unknown) are shown in different colors and line styles. *PiiL* allows for checking functional annotations for any gene in the pathway by loading information from GeneCards (http://www.genecards.org), NCBI Pubmed (http://www.ncbi.nlm.nih.gov/pubmed), or Ensembl (http://www.ensembl.org) into a web browser through one click.

### Highlighting DNAm level differences

DNAm data is read with CpG sites as the rows, and beta values (estimate of methylation level using ratio of intensities between methylated and unmethylated alleles) in the columns. *PiiL* accepts data from whole genome bisulfite sequencing (Bismark [30] coverage files), as well as any of Illumina’s Infinium methylation arrays (HumanMethylation27 BeadChip, HumanMethylation450 BeadChip or MethylationEPIC BeadChip). In any of the input formats, the CpG/probe IDs or positions need to be replaced with their annotated gene name. A Java application named *PiiLer*, also distributed with the software, uses pre-annotated files (done by Annovar [31]), to perform the conversion.

Genes are colored on a gradient from blue for low methylation levels (beta-value or methylation percentage), through white (for methylation level close to 0.5) to red when methylation levels approach 1. Once loaded, the metadata can be reused in different pathways.

**Fig. 1:**
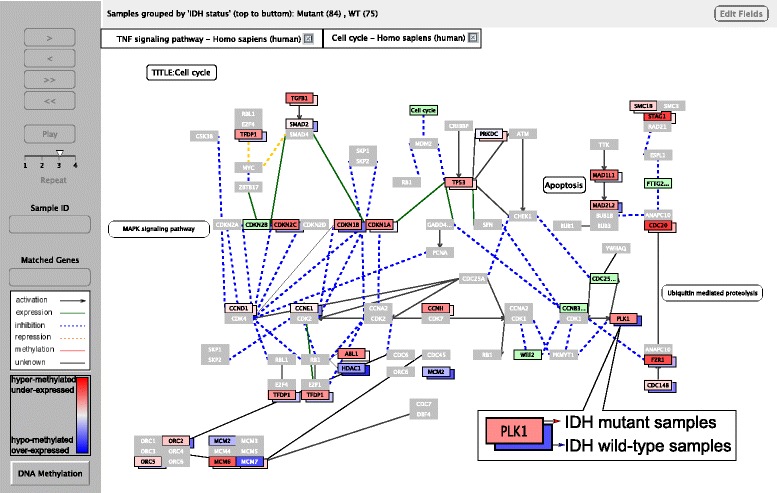
A snapshot of *PiiL* in group-wise view mode, showing the “cell cycle” pathway. Samples are grouped by IDH mutation status, each group represented by a box for each gene. The average of beta values of each group defines the color ranging from dark blue (unmethylated) through white (half-half) to dark red (methylated). The number of samples in each group is shown in parenthesis on top of the pathway panel. The panel on the left allows for navigating through the samples. The genes in light green did not get any match from the loaded data and the ones in gray do not have any CpG site based on the applied filter

Since there are typically multiple CpG sites per gene, additional information, such as the CpG ID, genomic position, and genomic location relative to a gene (for example intronic, exonic, upstream, UTR5, etc.) can be added to the gene name (separated with an underscore), allowing to quickly group sites by location and putative function. In this case, the methylation levels of all sites are averaged to set the color, and the gene border is colored green as an indication. The methylation status of each of the multiple sites hitting a gene can be viewed in a pop up window allowing the user to select or deselect specific sites to be included/excluded in the analysis. Figure 1 shows a snapshot of the *PiiL* screen.

### Selecting a subset of CpG sites

*PiiL* allows for selecting a subset of CpG sites to be included in the analysis (i.e. for assigning the color for a specific gene, producing plots and etc.). There are multiple options for including/excluding specific CpG sites:

- Filtering out the CpG sites that have very little variation by choosing a threshold for the standard deviation of the beta values for each site over all samples.
- Selecting CpG sites based on user defined ranges for beta values.
- Selecting CpG sites based on their annotated genomic position. For example, selecting the CpG sites that are exonic, UTR5, etc.
- Providing a list of pre-selected CpG sites with the CpG ID or genomic position.

These functions facilitate the visibility of the difference between the methylation levels of different groups of samples. Since averaging the beta values of all sites including the ones that do not vary significantly between the samples for color-coding, the differentiating signal is weakened and often difficult to detect. The genes with no CpG site present on the list of selected sites or no site passing the standard deviation filtering criteria are colored in gray.

### Highlighting gene expression level differences

FPKM (Fragments Per Kilobase of transcript per Million mapped reads) gene expression values are the second type of metadata that can be loaded into a pathway. Genes are colored for each sample according to the log2-fold difference between the expression value of the current sample and the median of expression values of all samples. The user can set the difference scale; by default, ranging from −4 to +4. To make colors comparable with DNAm beta values, the n-fold over-expressed genes are colored in *blue*, and the n-fold under-expressed ones are colored in *red*, with white indicating little or no differences. We note that this color convention is inverse to expression-centric color schemes, but greatly facilitates finding patterns that are shared between DNAm and expression in case higher methylation correlates with lower or silenced expression.

### Different view modes

There are three different view modes for reviewing the data and highlighting potential patterns: 1) single-sample view, 2) multiple-sample view and 3) group-wise view, where the median methylation/expression level is shown for each group of samples. More details can be found in the Additional file 1: S1.

### Finding similar-patterns

The “find similar-patterns” function allows for mining for genes with similar or dissimilar patterns of methylation or expression to any given gene or set of CpG sites, based on the Euclidian distance (check Additional file 1: S2).

### Browsing pathway independent genes

Genes that are not part of any known pathway can be displayed in a grid of genes, termed *PiiLgrid*. While not constituting a connected pathway, all functionalities of *PiiL* are also applicable to that set of genes. This option is useful after finding the genes with identical methylation pattern to a targeted gene. The set of genes can be browsed in a new tab for further analysis, for example, comparing their expression level with the targeted gene.

### General functions

For both methylation and expression values, the metadata over all samples can be viewed as a bar plot or histogram for each gene. In group-wise view, the members of each group are shown in the plots. Pathways, color-coded metadata and all the plots generated by *PiiL* can be exported to vector quality images in all viewing modes, which can be used in posters or publications. The manual page is accessible directly from the tool and users can send their feedback via the options in the tool. An option is provided to check for the latest available version and provides a downloadable runnable file of the latest version.

After checking multiple files in different pathways, a summary can be generated reporting the file name and the pathway that it was checked against followed by the list of matched genes.

## Results

“Glioma” refers to all tumors that originate from glial cells, non-neuronal cells that support neuronal cells in the brain and nervous system. Gliomas are classified by the World Health Organization (WHO) as grades I to IV [32, 33]. Lower Grade Gliomas (LGG) comprises diffuse low-grade and intermediate-grade gliomas (WHO grades II and III), with a survival ranging widely from 1 to 15 years [34]. Glioblastoma multiform (GBM), also known as astrocytoma WHO grade IV, is the most common type of glial tumors in humans, and also the most fatal brain tumor with a median survival time of 15 months [35]. A recent study, however, reported this classification as obsolete. They identified a different grouping that is based on mutations in the IDH1 and IDH2 genes, which allows for a more accurate classification [14]. To examine the possible downstream effects in more depth, we extracted 65 and 100 samples with GBM and LGG from the TCGA (The Cancer Genome Atlas) datasets accordingly [34, 36], for which both methylation (profiled using Illumina’s HumanMethylation450 BeadChip) and expression data are available (https://gdc-portal.nci.nih.gov/legacy-archive/search/f).

### Pathways at a glance

**Fig. 2:**
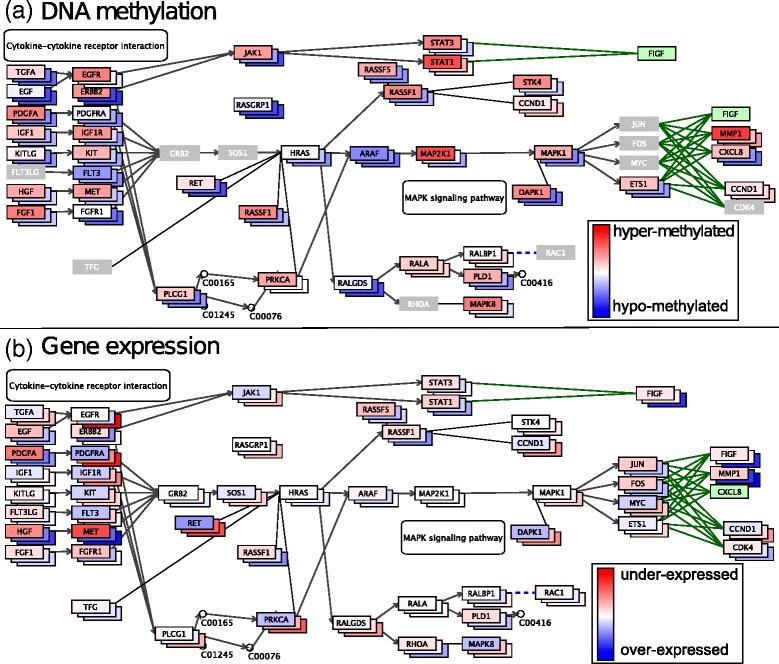
The “cytokine-cytokine receptor interaction” subsection of the “pathways in cancer” pathway, with (**a**) DNA methylation data, and (**b**) gene expression data. Samples are grouped by IDH mutation status (mutant, wild type, unknown). For the methylation data, we applied a standard deviation filter of >0.2, rendering genes in gray if they are devoid of passing sites. For each gene, the three boxes are colored according to the average methylation (**a**) or median expression (**b**)

**Fig. 3:**
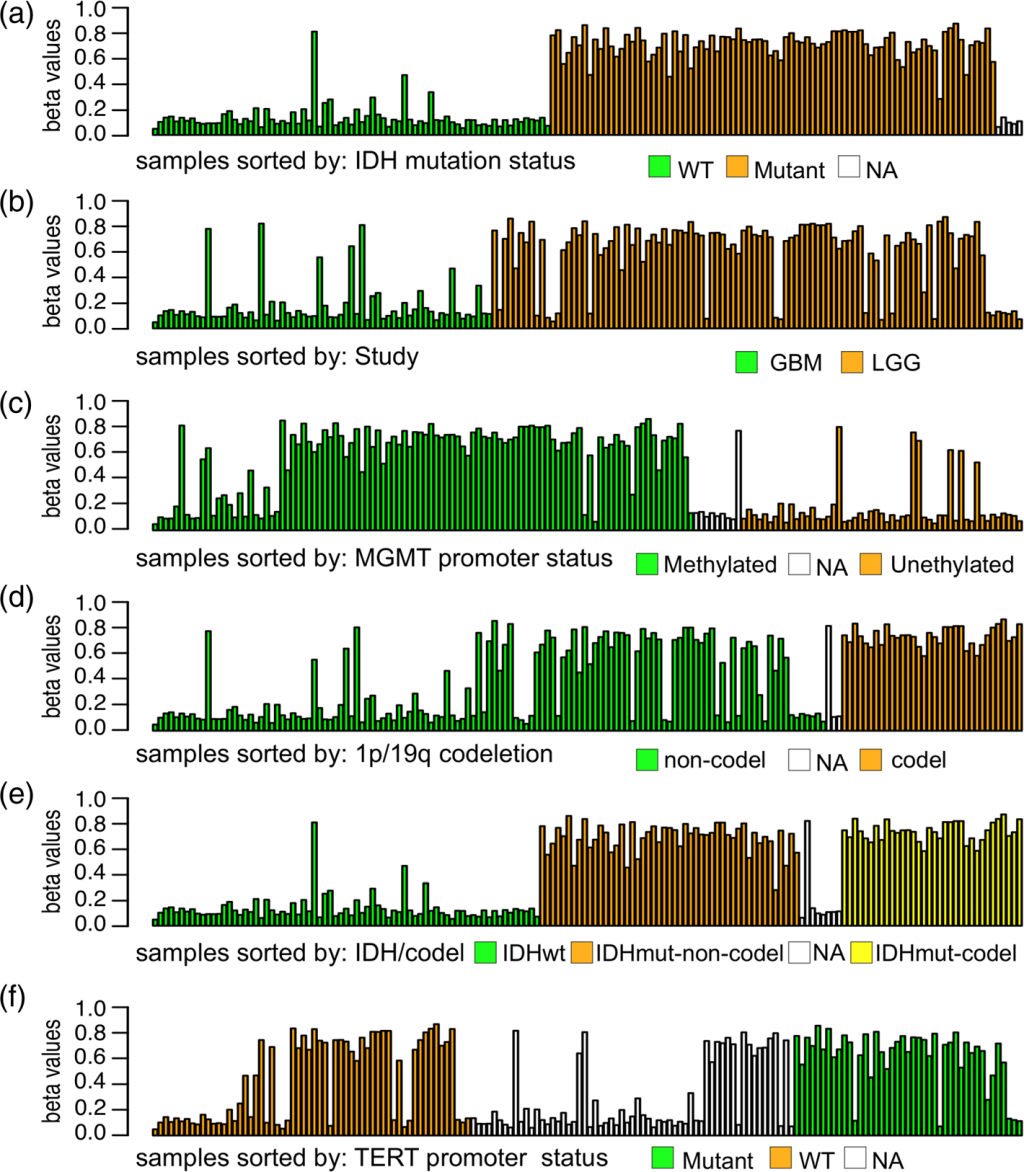
Bar plots of beta values of all samples grouped by different metadata categories: **a** IDH mutation status, **b** histology, **c** MGMT promoter status, **d** codeletion of 1p and 19q arms, **e** IDH mutation status together with codeletion of 1p and 19q arms, and **f** TERT promoter status

For a first assessment of the data, we examined the “cytokine-cytokine receptor interaction” subsection of the “pathways in cancer” expression network from KEGG (Fig. 2a), showing methylation of CpG sites that exhibit a standard deviation of more than 0.2 across all 165 samples, and grouping the data by IDH mutation status, i.e. wild-type, mutant, or unknown. Several genes are associated with CpG sites that drastically differ in methylation, shown in dark blue (unmethylated) and dark red (methylated), among them, *ERBB2*, a member of the epidermal growth factor (*EGF*) family and known to be associated with glioma susceptibility [37–40]. Gene expression of *ERBB2* is also altered and 2-fold lower in the IDH mutant samples, as shown in dark red (Fig. 2b). We next examined methylation values across samples using the bar plot view feature and using different groupings according to recorded phenotypes or molecular alterations in Glioma studied by [41] (Fig. 3). Here, we can visually confirm that the mutation status of IDH is the best predictor for methylation (Fig. 3a). In addition, all samples without known IDH status are lowly methylated and could thus be putatively classified as ‘wild-type’. By contrast, codeletion of chromosome arms 1p and 19q (1p/19q codeletion), reported to be associated with improved prognosis and therapy in low-grade gliomas patients [42], appears to have no effect on the methylation of *ERBB2*. Likewise, neither mutations in the promoter of the *TERT* (Telomerase Reverse Transcriptase) gene [41], nor the promoter methylation status of the gene encoding for repair enzyme *O6-methylguanine-DNA methyltransferase* (*MGMT*), which has been reported to be correlated with long-term survival in glioblastoma [43, 44], plays an obvious role in the methylation of this and other genes in the pathway.

**Fig. 4:**
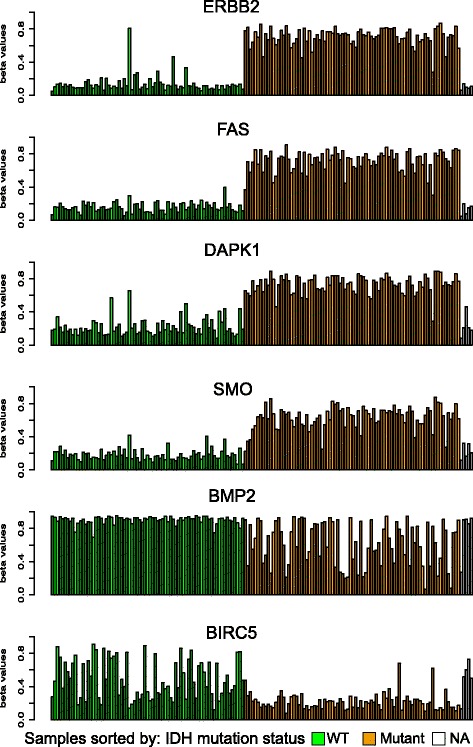
Bar plot of beta values of all samples for gene *ERBB2* compared with *FAS*, *DAPK1*, and *SMO*, which contain sites most similar in methylation. Shown are also *BMP2* and *BIRC5*, which are associated with sites most dissimilar. Samples are grouped by IDH mutation status

For an overall survey of how many genes exhibit methylation patterns similar to *ERBB2*, we applied *PiiL’s* “find similar-patterns” feature, listing genes with the least Euclidian distance of beta values. The top three genes (Fig. 4) with the most similar patterns are *FAS*, a gene with a central role in the physiological regulation of programmed cell death; *DAPK1*, Calcium/calmodulin-dependent serine/threonine kinase involved in multiple cellular signaling pathways that trigger cell survival, apoptosis, and autophagy; and *SMO*, G protein-coupled receptor that probably associates with the patched protein (PTCH) to transduce the hedgehog proteins signal (http://www.genecards.org). There, we found that in *FAS, SMO* and *ERBB2*, the average expression level of the samples in IDH mutants is lower than the average expression level of the wild-type samples, while for *DAPK1* the mutants exhibit higher expression levels. On the other end of the scale, *BMP2* and *BIRC5* host sites with the most distant pattern to *ERBB2* (Fig. 4). *BIRC5* is a member of the inhibitor of apoptosis gene family, negatively regulating proteins involved in apoptotic cell death (genecards.org). *BMP2* is a member of transforming growth factor superfamily with a regulatory role in adult tissue homeostasis, reported to be significantly down-regulated in recurrent metastases compared to non-metastatic colorectal cancer [45]. Interestingly, expression of *BMP2* is suppressed in wild type and unknown IDH status cancers, but high in some mutant samples in this data set.

### DNA methylation and gene expression

**Fig. 5:**
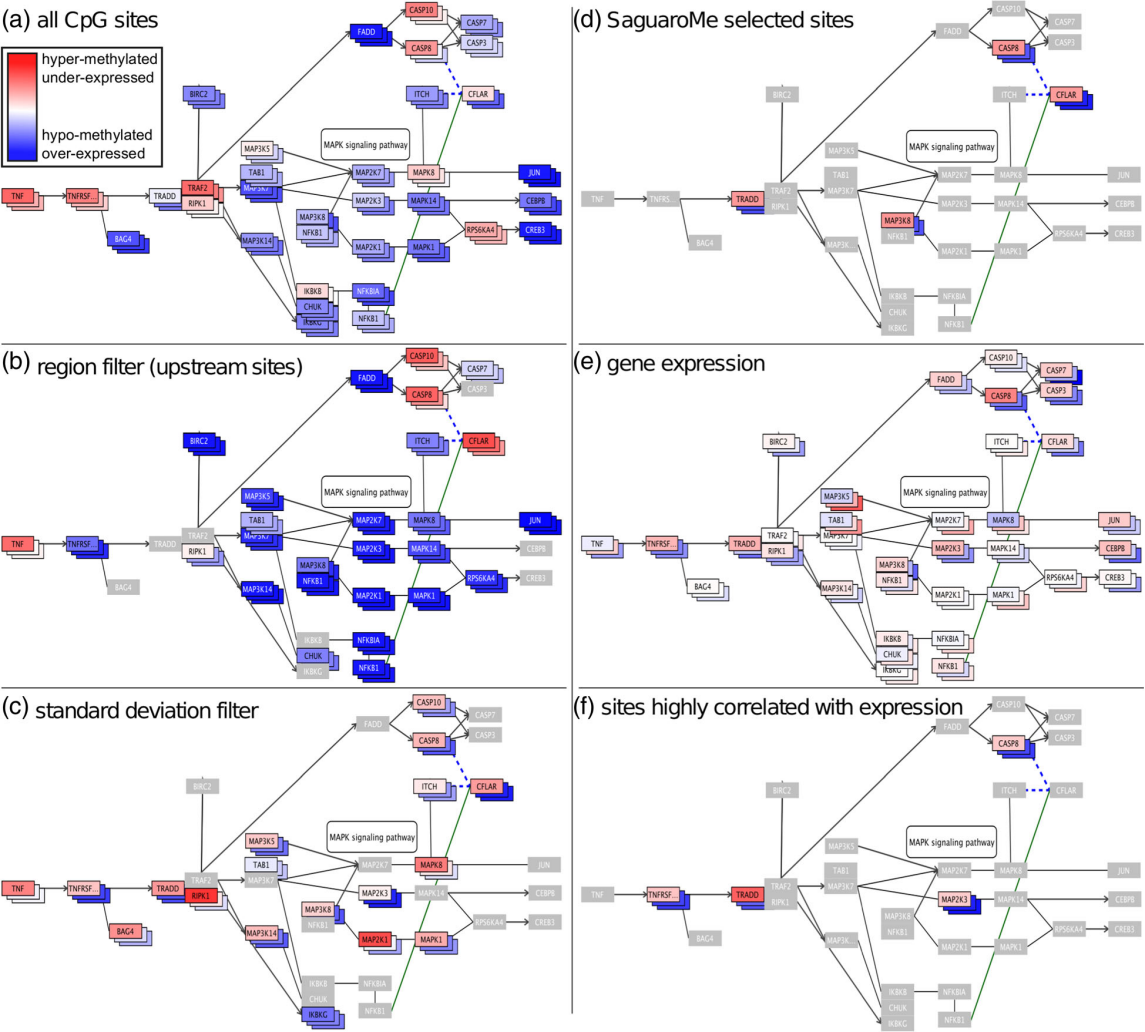
Effect of selecting different subsets of CpG sites in the “TNF signaling pathway”. Genes colored in gray do not contain any passing CpG sites. Filtering criteria are: (**a**) all CpG sites; (**b**) sites located at upstream or in the UTR5 of a gene; (**c**) sites with a standard deviation >0.2; (**d**) sites identified by Saguaro; (**e**) Gene expression; (**f**) sites with a high inverse correlation between methylation and expression. For each gene, the three boxes are colored according to average methylation (**a**-**d** and **f**) or median expression (**e**)

To demonstrate the effect of selecting different subsets of CpG sites, we examined both *PiiL’s* filters, as well as other DNAm analysis methods (Fig. 5). We first applied the unsupervised classification software Saguaro [21] to all CpG sites, detecting one pattern that perfectly coincides with IDH mutation status. Overall, genes with at least 10 CpG sites include *MYADM, CFLAR, PAX6, FRMD4A, MEIS1, TNXB, MACROD1, CHST8, SRRM3, CPQ, TBR1, SYT6, RNF39, ISLR2, EML2, BCAT1*, *ACTA1*, and, confirming results from our earlier visual inspection, *ERBB2*, which we examined earlier. The top pathways these genes are a part of include “pathways in cancer”, “mTOR signaling”, and “TNF signaling”. For the latter, we show the average methylation over all sites of all genes (Fig. 5a) and sites located upstream (Fig. 5b). Figure 5c shows the sites with a standard deviation smaller than 0.2, coloring genes without sites in light gray. The sites and genes identified by Saguaro (Fig. 5d); the log-fold changes in expression (Fig. 5e), and genes with sites exhibiting Speaman’s correlation < −0.7 between methylation and expression (Fig. 5d) are also shown for comparison.

Throughout this progression, we note that methylation values already change dramatically, mostly increasing, but in some cases decreasing, e.g. *TNFRSF*. In terms of correlation, we found four genes in this pathway at Spearman’s rank correlation coefficient (rho) > 0.7, *TNFRSF, TRADD, MAP2K3*, and *CASP8*, (Fig. 5f) for which hypermethylation of the promoter has previously been reported [46]. Two of these genes coincide with Saguaro, which reports two additional genes, *CFLAR* and *MAP3K8*, but not *TNFRSF* and *MAP2K3* (Fig. 5d).

### Methylation blocks expression in pathways

**Fig. 6:**
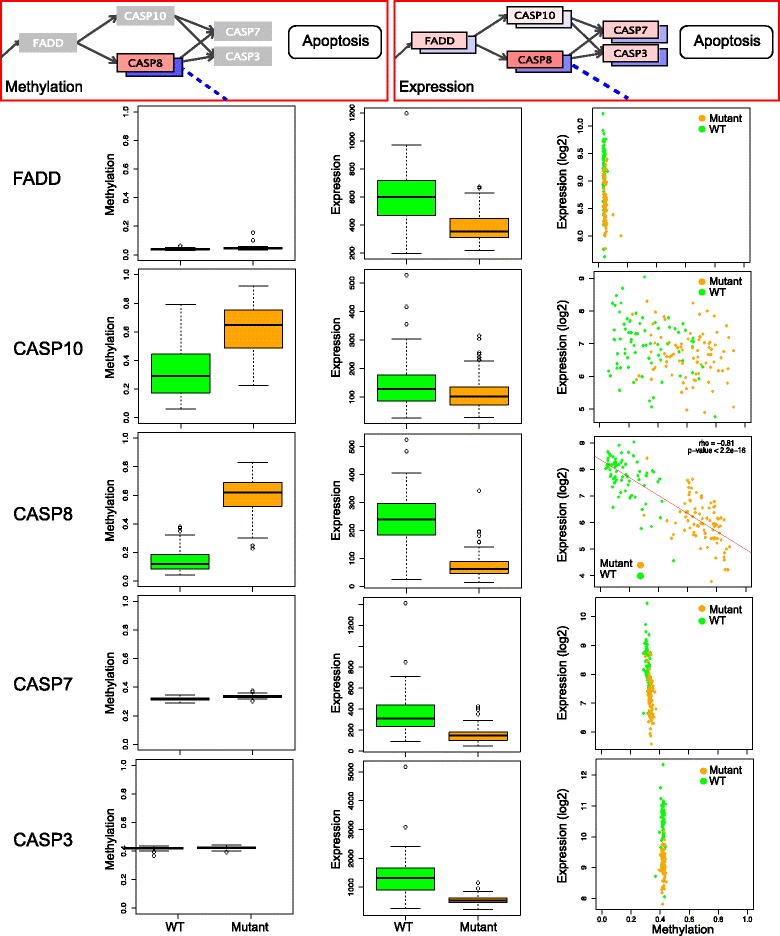
A subsection of the “TNF signaling pathway” leading into the apoptosis pathway, showing genes *FADD, CASP10, CASP8, CASP7* and *CASP3. FADD, CASP7* and *CASP3* are not subject to DNAm changes when comparing IDH wildtype to mutants. CASP10 exhibits somewhat higher methylation levels, but remains stable at expression. By contrast, sites selected by Saguaro in *CASP8* exhibit higher methylation levels in mutants, as well a correlated downregulation of expression. *CASP3* and *CASP7*, which are downstream of the CASCADE from CASP8, are almost entirely silenced in expression

Figure 6 shows the downstream part of the TNF signaling pathway that regulates or initiates the apoptosis pathway, consisting of *FADD, CASP8* and *CASP10*, which regulates *CASP7* and *CASP3*. Sequential cascade-like activation of caspases plays a central role in activating apoptosis, and both *CASP3* and *CASP7* appear downregulated or almost silenced. While both *CASP10* and *CASP8* are affected by changes in methylation, the beta values increase from less than 0.2 to more than 0.7 in *CASP8* in the CpG sites selected by Saguaro. In addition, expression is highly negatively correlated with methylation (Spearman’s rho = −0.81, *p*-value <2*10^−16^), suggesting that *CASP8* acts as the blocking factor in the expression cascade. None of *CASP3, CASP7* or *FADD,* which are situated upstream in the pathway, are differentially methylated, and the decreased expression of FADD can possibly be explained by differential methylation/expression of the upstream *TRADD* gene.

An alternative way to visualize changes in a large number of samples is implemented in *PiiL*’s ‘playback’ feature. After sorting the samples by methylation of *CASP8* in increasing order, Additional file 2: Video S1 shows methylation and expression in the TNF signaling pathway, rendering *TRADD*, *CASP8*, *CFLAR* and *MAP3K8* dark blue in the beginning (low methylation), and then sharply turning red when switching from showing wild type samples to IDH mutant samples. Expression changes follow methylation but more loosely, with several genes appearing blue (high expression) in the beginning, and transitioning to red (low expression) later on, as shown side by side with methylation in Additional file 2: Video S1.


Additional file 2: Video S1. *PiiL* showing DNA methylation and gene expression along samples. Color-coded data of all samples is shown consecutively using *PiiL’s* playback feature. For each gene, the box on top shows methylation, and the box behind shows gene expression. For the methylation data, genes with sites selected by Saguaro are highlighted. The samples are sorted according to the ascending order of beta values for the *CASP8* gene. (MP4 837 kb)


### Genes inside and outside of known pathways

**Fig. 7:**
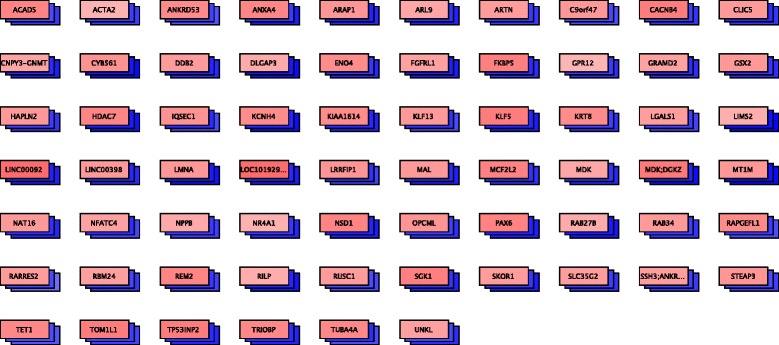
A *PiiLgrid* generated for genes covering CpG sites with a similar methylation pattern to ERBB2, showing CpG sites with a standard deviation over 0.2

Changes in methylation and expression can affect many genes, a large fraction of which may not be members of known pathways. To provide all analysis and visualization features for these genes as well, *PiiL* implements the “PiiLgrid” feature, which allows to display a any set of genes regardless of the pathway, but giving access to all analysis features. An example, genes that harbor sites similar to *ERBB2*, is shown in Fig. 7.

## Conclusions

Advances in RNA and DNA sequencing allow for generating large amounts of RNA expression and DNA methylation data. Following the relatively inexpensive DNAm Bead Chip for human studies, we anticipate that genome-wide bisulfite sequencing will add more data and for a number of different organisms. While tools and methods for analyzing differential methylation and expression exist, any functional interpretation is best understood when integrating and visualizing the data in context of expression networks or pathways. *PiiL* is a browser for DNAm and RNA-Seq data, allowing direct comparison and testing specific hypotheses, in particular in model organisms for which pathway and expression network data exists. Its integrated analysis features provide the ability to quickly assess large amounts of data points, genes, and CpG sites, and navigating within and between pathways. Using the publicly available glioma data set, we have shown that a rich set of interesting aspects about this data is accessible with a few mouse clicks and within a few minutes. We thus anticipate that *PiiL*, and perhaps other interactive visualization tools, will be as common and widely used for epigenomic analyses as genome browsers are today for genomic analyses.

## Additional files

Additional file 1: S1. View Modes. **S2.** Calculating the Euclidian distance. (DOCX 90 kb)

## Abbreviations

GBM: Glioblastoma Multiform
IDH: Isocitrate dehydrogenase
KEGG: Kyoto Encyclopedia of Genes and Genomes
LGG: Lower Grade Glioma
TCGA: The Cancer Genome Atlas
