# Supplementary material for: PiiL: visualization of DNA methylation and gene expression data in gene pathways
Source: BMC Genomics. 2017 Aug 2;18:571. doi: 10.1186/s12864-017-3950-9 (PMC5541427; doi:10.1186/s12864-017-3950-9)
Supplement: Supplementary file 2 — View Modes. S2. Calculating the Euclidian distance. (DOCX 90 kb) [file 12864_2017_3950_MOESM1_ESM.docx]

**S1. View Modes**

There are three different view modes for reviewing the data and highlighting potential patterns:

**1) Single-sample view**

The user can navigate through the metadata in an animation mode, where each sample represents one frame. Playback speed is adjustable, and frames can be stepped through manually as well. In case of DNAm data, the user can navigate through the samples and check the status of all the sites hitting a selected gene. The samples’ descriptive information can be also loaded and displayed while navigating through the samples. This makes the characteristics of a sample that might show interesting patterns easy to review. This information can also be used for grouping the samples based on one of their provided features (see group-wise view).

**2) Multiple-sample view**

In this mode, the status of each gene is shown in a static view as stacked boxes for 10 consecutive samples. This view enhances comparing the patterns of methylation or expression of multiple samples simultaneously for all, or a selection of genes in the pathway.

**3) Group-wise view**

Samples can be grouped by one of the columns of the samples information file. The group-wise view shows the median methylation/expression level of the samples of each group.

There are two options for grouping gene expression:

1) Comparing the median expression value of the samples of each group to the median of all samples.

2) Comparing the median expression value of the samples of each group, with the median of the samples belonging to a group specified as the “base group”.

**S2. Calculating the Euclidian distance**

For DNAm, the beta values of the selected sites of the targeted gene are averaged and then searched against all CpG sites in the data set or the genes in the active tab. For example if there are *m* samples the distance between gene *A* and gene *B* is defined as:

$$\sum_{i=1}^{m} \left( A_{i}-B_{i} \right)^{2}$$

For expression, FPKM values are normalized for absolute expression, and then compared across genes. In either case, the targeted gene can be compared with methylation of all the CpG sites or expression of all genes in the whole loaded metadata file.

The genes and their CpG sites will be sorted according to their Euclidian distance of the genes for each sample.
